# Proteinuria as a presenting sign of combined methylmalonic acidemia and homocysteinemia: case report

**DOI:** 10.1186/s12881-020-01122-x

**Published:** 2020-09-21

**Authors:** Ru-Yue Chen, Xiao-Zhong Li, Qiang Lin, Yun Zhu, Yun-Yan Shen, Qin-Ying Xu, Xue-Ming Zhu, Lin-Qi Chen, Hai-Ying Wu, Xu-Qin Chen

**Affiliations:** 1 Department of Nephrology and Immunology, Children’s Hospital of Soochow University, Suzhou, Jiangsu China; 2 Department of Pathology, Children’s Hospital of Soochow University, Suzhou, Jiangsu China; 3 Department of Endocrinology, Children’s Hospital of Soochow University, Suzhou, Jiangsu China; 4 Department of Neurology, Children’s Hospital of Soochow University, Suzhou, Jiangsu China

**Keywords:** Children, Methylmalonic acidemia(MMA), Homocysteinemia, Proteinuria, Vitamin B12

## Abstract

**Background:**

Disorders of the metabolism and absorption of vitamin B12 can lead to decrease in activity of methionine synthetase and methylmalonate coenzyme A mutase (MMUT), which results in increased levels of methylmalonic acid and homocysteine in blood and urine. Often, combined methylmalonic acidemia (MMA) and homocysteinemia is misdiagnosed due to a lack of specific symptoms. The clinical manifestations are diverse, but proteinuria as the initial presentation is rare.

**Case presentation:**

Two cases of MMA with homocysteinemia in children are reported. Proteinuria were a primary presenting symptom, followed by anemia and neurologic symptoms (frequent convulsions and unstable walking, respectively). Screening of amino acids and acyl carnitine in serum showed that the propionyl carnitine:acetylcarnitine ratio increased. Profiling of urinary organic acids by gas chromatography–mass spectrometry revealed high levels of methylmalonic acid. Homocysteine content in blood was increased. Comprehensive genetic analyses of peripheral blood-derived DNA demonstrated heterozygous variants of methylmalonic aciduria type C and homocystinuria (*MMACHC*) and amnionless (*AMN*) genes in our two patients, respectively. After active treatment, the clinical manifestations in Case 1 were relieved and urinary protein ceased to be observed; Case 2 had persistent proteinuria and was lost to follow-up.

**Conclusions:**

Analyses of the organic acids in blood and urine suggested MMA combined with homocysteinemia. In such diseases, reports of renal damage are uncommon and proteinuria as the initial presentation is rare. Molecular analysis indicated two different genetic causes. Although the pathologic mechanisms were related to vitamin B12, the severity and prognosis of renal lesions were different. Therefore, gene detection provides new insights into inherited metabolic diseases.

## Background

Methylmalonic acidemia (MMA) involves abnormal metabolism of amino acids and organic acids. It is caused mainly by abnormal accumulation of metabolites such as methylmalonic acid, 3-hydroxypropionic acid, and methyl citric acid due to deficiency of methylmalonate coenzyme A mutase (MMUT) or a defect in the metabolism of the coenzyme cobalamin (Cbl). Cbl (also known as vitamin B12) has four bioactive forms and several analogs [1]. Cyanocobalamin (CNCbl) is a stable and inexpensive synthetic form used commonly for food fortification and oral/parenteral supplementation [2]. Vitamin B12 is converted into active adenosylcobalamin (AdoCbl) and methylcobalamin (MeCbl) through enzymatic reaction in the cytoplasm and mitochondria mainly, and participates in the metabolism of amino acids [3]. AdoCbl is a cofactor for MMUT, and its deficiency leads to accumulation of methylmalonic acid and its precursors (e.g., propionic acid, methylcitric acid, and other metabolites) in the body, a decrease in activity of succinate dehydrogenase, and obstruction of generation of mitochondrial energy. MeCbl is a cofactor for methionine synthetase, which catalyzes the re-methylation of homocysteine to methionine. A lack of methionine synthetase leads to homocysteine accumulation and consumption of methionine and S-adenosylmethionine within the cell [4–6]. The clinical manifestations are diverse, and more common in blood and nervous system [7, 8]. Secondary renal damage has also been reported, but proteinuria as the primary presenting symptom is rare. Here, we report two cases of MMA with homocysteinemia in children with proteinuria as a presenting sign. The genetic-mutation sites were different, as were the pathogenesis and prognosis of the disease.

## Case presentation

### Case 1

**Fig. 1:**
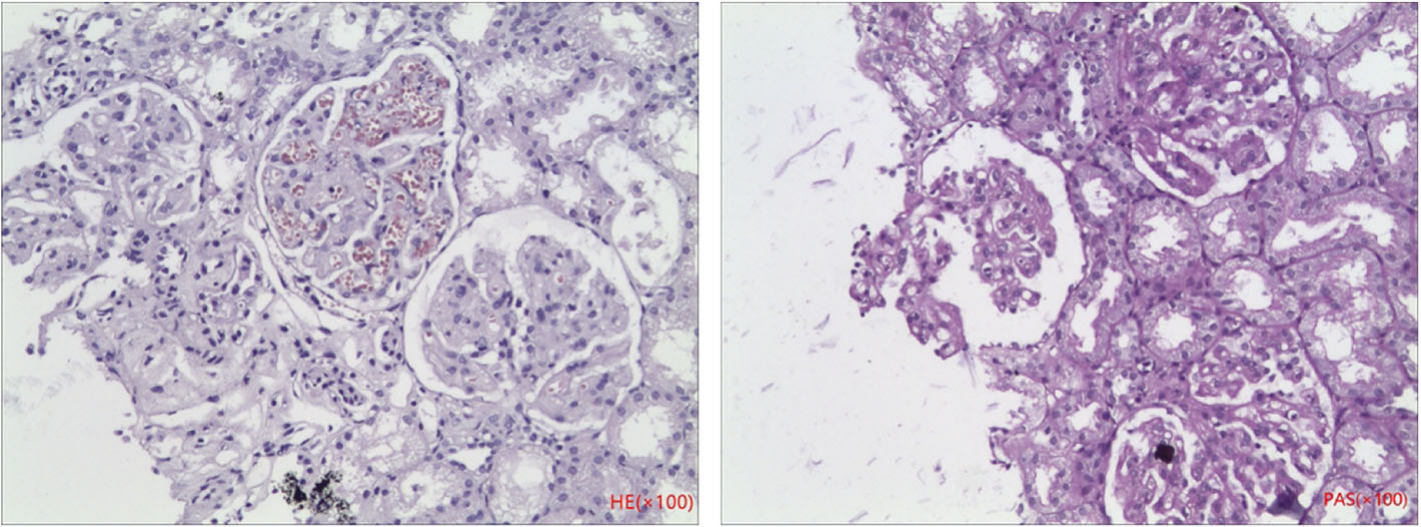
Renal biopsy under light microscopy. Diffuse membranoproliferative lesions of the glomerulus with changes in congestion of glomerular capillary loops, some degeneration of tubular epithelial cells, and intraluminal protein casts

**Fig. 2:**
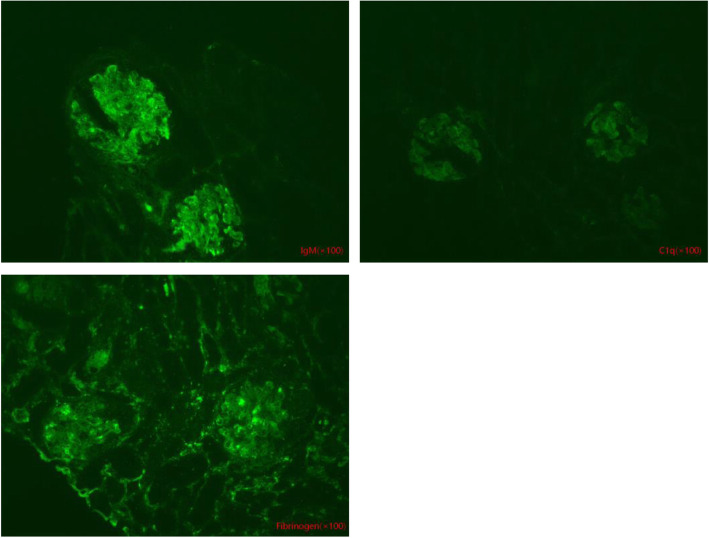
Immunofluorescence analysis of renal biopsy. Diffuse granular deposition of IgM (3+), C1q (+) and fibrinogen (+) along the glomerular mesangium and capillary loops

**Fig. 3:**
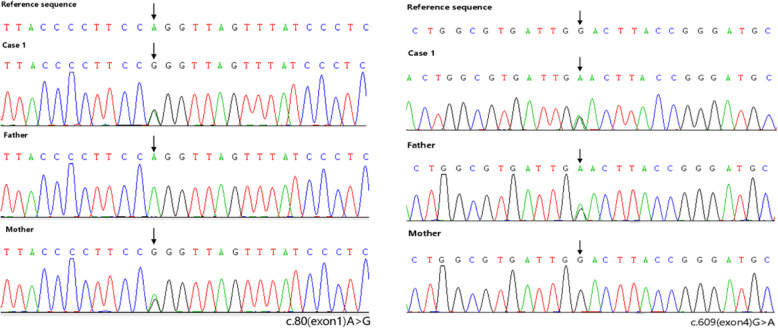
Gene sequences of Case 1 and his parents

A 42-month-old Chinese boy (bodyweight, 16 kg; height, 102 cm) was admitted to the Department of Nephrology and Immunology of our hospital due to edema and proteinuria. He underwent laboratory examination for macrocytic anemia (hemoglobin, 58 g/L), proteinuria (2+) and hematuria (103/HP). The 24-h urinary level of microprotein increased to 75.7 mg/24 h (reference range: 2.6–16.6 mg/24 h). Anemia, proteinuria, and hematuria continued after infusion of washed red blood cells (RBCs), methylprednisolone administration, and anti-infection treatment after hospital admission. Ten days later, he suddenly developed frequent convulsions and persistent hypertension. He was transferred to the intensive care unit for tracheal intubation, and methylprednisolone pulsation treatment (300 mg/d for 3 days followed by gradual reduction) and symptomatic treatment. The convulsions ceased and hypertension was relieved, but anemia, proteinuria, and hematuria persisted. Renal biopsy was done. Under light microscopy, diffuse membranoproliferative lesions of the glomerulus were noted. Also, changes in congestion of glomerular capillary loops, some degeneration of tubular epithelial cells, and intraluminal protein casts were documented (Fig. 1). Immunofluorescence analyses showed diffuse granular deposition of immunoglobulin M (IgM; 3+), C1q (+) and fibrinogen (+) along the glomerular mesangium and capillary loops (Fig. 2). Screening of amino acids and acyl carnitine in blood showed that the propionyl carnitine:acetylcarnitine ratio increased to 0.29 (reference range: 0.02–0.25). Also, profiling of organic acids in urine by gas chromatography–mass spectrometry revealed increased levels (in μmol/L) of methylmalonic acid (81.9; reference range: 0.0–4.0) and methyl citrate (0.9; 0.0–0.8). Further detection suggested the homocysteine content in blood to be > 100 μmol/L (reference range: 0.0–15.0 μmol/L). The level of vitamin B12 was normal (263.4 pg/mL; reference range: 200.0–900.0 pg/mL). Comprehensive genetic analyses of peripheral blood-derived DNA revealed compound heterozygous variants of the methylmalonic aciduria type C and homocystinuria (*MMACHC*) gene, which suggested the diagnosis was Methylmalonic Acidemia and Homocystinuria (cblC type) (Table 1; Fig. 3).

**Table 1:** Genetic testing of Case 1 and his parents

Gene	*MMACHC*	
Chromosomal location	Chr1:45974647	Chr1:45966084
Nucleotide	NM_015560. c.609(exon4)G > A	NM_015560. c.80(exon1)A > G
Amino acid	NM_015560. p.W203X,80(p.Trp203stop,80)	NM_015560. p.Q27R(p.Gln27Arg)
Maternal genotype	Wild type	Heterozygous
Paternal genotype	Heterozygous	Wild type
Diagnosis	Methylmalonic Acidemia and Homocystinuria (cblC type)

Treatment was initiated with betaine (4.5 g/day, p.o.), calcium folinate (5 mg/day, p.o.), methionine (0.2 g/day, p.o.), vitamin B12 (1 mg/day, i.m.), L-carnitine (1.5 g/day, p.o.) as well as feeding with standard milk powder:milk powder lacking methionine, threonine, valine and isoleucine at a protein ratio of 1:1.

The hemoglobin level increased gradually, blood pressure remained stable, and metabolites in blood and urine was essentially normal. However, proteinuria and hematuria persisted.

The treatment stated above was combined with mycophenolate mofetil (MMF; 250 mg/day, p.o.). After 3 months, proteinuria and hematuria reversed. The patient had regular follow-up in the outpatient department and medication adjustment according to follow-up results.

### Case 2

A Chinese male aged 16 years and 5 months (bodyweight, 89 kg; height, 169 cm) visited the Department of Neurology in our hospital because of unstable walking of 2-week duration. When he was 2 years of age, he had been sent to a local hospital for examination because of unwillingness to walk. Laboratory tests revealed megaloblastic anemia combined with proteinuria. Renal biopsy had shown isolated proteinuria (specific disease not known) but treatment had not been initiated. Anemia and proteinuria persisted.

After hospital admission, he was treated with neurotrophic drugs, but symptoms were not alleviated. Routine, biochemical, culture and autoimmune encephalitis antibody in cerebrospinal fluid were normal. Electroencephalography was normal. The T1-weighted imaging (T1WI) sequence of cranial magnetic resonance imaging (MRI) showed decreased signals for the clivus and cranial barrier. T1WI and T2WI sequences of MRI showed uniform reduction of signals in the whole spinal column, centrum and accessories. Screening for amino acids and acyl carnitine using blood samples revealed the level of propionyl carnitine (15.46 μmol/L; reference range: 0.38–3.6 μmol/L) and the ratio of propionyl carnitine:acetylcarnitine (0.48; reference range: 0.04–0.22) to be increased. Also, profiling of urinary organic acids by gas chromatography–mass spectrometry showed high levels (in μmol/L) of 3-hydroxypropionic acid (13.07; reference range: 0.0–1.1) and methylmalonic acid (50.82; 0.2–3.6). The homocysteine concentration in serum was 85.6 μmol/L (reference range: 0.0–15.0 μmol/L). The level of vitamin B12 was the lower limit of normal (231.2 pg/mL; reference range: 200.0–900.0 pg/mL). Genetic analyses of peripheral blood-derived DNA was undertaken and showed compound heterozygous variants of the amnionless (*AMN*) gene (Table 2) and suggested the diagnosis of Imerslund–Gräsbeck syndrome (IGS). After 1 week of treatment comprising L-carnitine (3 g/day, p.o.), vitamin B12 (1 mg/day, i.m.), vitamin B6 (30 mg/day, p.o.), folic acid (5 mg/day, p.o.), coenzyme Q10 (10 mg/day, p.o.) and betaine (3 g/day, p.o.), the level of homocysteine in blood and methylmalonic acid in urine decreased to normal ranges. The hemoglobin level increased gradually and remained stable, but proteinuria and unsteady walking persisted. The amino acids and other metabolites in blood and urine were measured regularly, and the dose and frequency of drugs adjusted according to the results. After 4 months, the patient still had proteinuria (2+) and then was lost to follow-up.

**Table 2:** Genetic testing of Case 2 and his parents

Gene	*AMN*	
Chromosomal location	Chr14: 103395992	Chr14: 103395855
Nucleotide	NM_030943. c.761G > A	NM_030943. c.742C > T
Amino acid	NM_030943. p.G254E	NM_030943. p.Q248*
Maternal genotype	Wild type	Heterozygous
Paternal genotype	Heterozygous	Wild type
Diagnosis	Imerslund–Gräsbeck syndrome	

## Discussion and conclusions

Disorders of inborn errors of cobalamin metabolism is an inherited metabolic disease.

Various factors lead to MMA, where it is biomarker for some inhirited metabolic disorders such as the deficiency of MMUT or coenzyme Cbl, which results in abnormal accumulation of metabolites however the latter can also cause homocysteinemia. The global prevalence of MMA has been reported to range between 0.0004 and 0.0021% [9]. The incidence of MMA in neonatal screening in China has been reported to be 0.0038–0.025%, and MMA with homocysteinemia is relatively common [9, 10].

### Methylmalonic Acidemia and Homocystinuria (cblC)

Combined MMA and homocysteinemia is an inherited metabolic disease related to a disorder of metabolism of vitamin B12. According to genetic defects, cblC, cblD, cblF, cblX and cblJ types have been found, and cblC appears to be more common [3, 10, 11]. Impaired synthesis of intracellular AdoCbl and MeCbl due to a *MMACHC* mutation located in the p34 coding region of staining 1 has been shown to reduce the activity of methionine synthase and MMUT, resulting in accumulation of methylmalonic acid and homocysteine *in vivo* [12, 13]. *MMACHC* has a length of 10,736 bp and contains four coding exons and one noncoding exon, and encodes a polypeptide of 282 amino acids [9]. In China, most variants are clustered in exons 3 and 4, and c.609G > A (p.W203X) is the most frequent cblC mutation [9, 14]. Case 1 presented with proteinuria, hematuria, megaloblastic anemia, frequent convulsions and persistent hypertension. Metabolite detection in blood and urine samples showed MMA combined with homocysteinemia. Further genetic analyses showed two heterozygous *MMACHC* variants, c.80A > G (p.Q27R, from his mother) and c.609G > A (p.W203X, from his father), which were consistent with cblC type.

### Imerslund-Gräsbeck syndrome (IGS)

IGS is a rare autosomal-recessive genetic disease characterized by poor absorption of Cbl within the intestine [15, 16]. Hereditary malabsorption of Cbl has been reported to involve three genes: variants of cubilin (*CUBN*) and/or *AMN* lead to IGS, and *GIF* variants lead to intrinsic factor deficiency (IFD). Clinically, the difference between IGS and IFD is that proteinuria is present in IGS [4, 17]. The endocytic receptor cubam is formed by the 460-kDa protein cubilin (gene product of *CUBN*) and the 45-kDa transmembrane protein amnionless (gene product of *AMN*). Cubam and amnionless are part of the intrinsic factor–cobalamin complex. The latter shows high expression in the distal intestinal tract and proximal renal tubules, and has important roles in the absorption of Cbl within the intestine and reabsorption of urinary protein in the kidney. Hence, a *CUBN* mutation on chromosome 10 and/or *AMN* on chromosome 14 can lead to IGS [18–20]. Genetic analyses of peripheral blood-derived DNA in Case 2 revealed two heterozygous variants of *AMN*, c.742C > T (p.Q248*, from his mother) and c.761G > A (p.G254E, from his father), in accordance with IGS. Namour F et al. reported a case with similar clinical manifestation and laboratory examination in France, in which genetic tests showed composite heterozygosity of c.742C > T, p.Gln248X and c.208-2A > G [18]. Also, c.761G > A (p.G254E) has been reported in other countries [17].

### Renal lesion

cblC involves abnormal metabolism of vitamin B12 and IGS involves malabsorption of vitamin B12. Both can lead to a decrease in the activity of methionine synthetase and MMUT, which results in increased levels of methylmalonic acid and homocysteine in blood and urine. The clinical manifestations of cblC can be divided into three periods: prenatal, infantile and non-infantile, in which infantile presentation is most common [12]. Hemolytic uremic syndrome (HUS) has been reported for the cblC type [21–24]. Homocysteine promotes the development of microvascular thrombosis by damaging endothelial cells, increased expression of procoagulants, induction of fibrinogen activation, and stimulation of pro-inflammatory signaling pathways, which are closely related to HUS in cblC [25]. Mathilde L et al. reported that most patients presented with renal thrombotic microangiopathy (TMA) with acute renal failure in cblC deficiency-associated kidney lesions, and suggested that all patients with renal TMA should be screened for metabolic disorders of Cbl [26]. Angel F et al. observed vitamin-B12 deficiency to be common among patients with hyper-homocysteinemia and thrombosis [27]. Renal biopsy in Case 1 showed glomerulopathy to be the main pathologic change. Specifically, diffuse membranoproliferative lesions of the glomerulus with deposition of IgM (3+), C1q (+) and fibrinogen (+) along the glomerular mesangium and capillary loops. IGS is associated with non-progressive proteinuria which, in general, does not affect renal function. Data from clinical trials have suggested that a reduction of urinary protein reabsorption due to defects in expression of cubilin and amnionless, followed by proteinuria, are not related to kidney disease [15, 28, 29]. Boina AA et al. [4] reported that, after treatment with vitamin B12 in children with IGS, the RBC count and metabolic parameters returned to normal, whereas proteinuria persisted. However, a *CUBN* mutation was found in a multicenter study of childhood corticosteroid-resistant nephrotic syndrome, and one case progressed to end-stage renal disease [30]. Some scholars have found that the homocysteine concentration in plasma decreases and the homocysteine level in urine increases in patients or models of nephrotic syndrome [31, 32], which is probably related to some direct urinary loss of homocysteine due to albumin binding. We suspect that the increased homocysteine level causing proteinuria in our cases may have been related to increased urinary excretion of albumin-bound homocysteine.

### Treatment and prognosis

The main treatment of MMA with homocysteinemia involves high-dose intramuscular injections of hydroxocobalamin, which can decrease the concentrations of homocysteine and methylmalonic acid, and increase the synthesis of methionine [12, 33]. At present, application of MeCbl and AdoCbl does not aid prevention/treatment of Cbl deficiency, and CNCbl is thought to be a more stable and inexpensive form and suited for oral supplementation and parenteral treatment [2, 12]. Betaine also enhances re-methylation of homocysteine to methionine, thereby reducing the level of homocysteine [31]. Levocarnitine can facilitates excretion of propionyl groups and prevents carnitine deficiency; levocarnitine can usually be given at 50–200 mg/kg/day [12]. Folic acid and other supplements, such as MeCbl and pyridoxine, are used as adjunct therapy. Protein restriction can be employed to lower the load of amino acids into the propionate oxidation pathway and decrease production of methylmalonic acid by partially blocking the MMUT step. However, such protein restriction induces deficiency of methionine and essential branched-chain amino acids, which can have negative effects on the growth and development of the brain [31]. In our cases, metabolite levels in blood and urine were essentially normal after active treatment of betaine, calcium folinate, methionine, vitamin B12, L-carnitine as well as dietary management. However, proteinuria persisted. MMF and glucocorticoid was applied in Case 1. About 3 months later, proteinuria reversed. He had a good prognosis, regular follow-up in the outpatient department, and medication adjustment according to follow-up results. However, Case 2 had persistent proteinuria and was lost to follow-up.

A disorder of the metabolism and absorption of Cbl can lead to MMA combined with homocysteinemia. The clinical manifestations are diverse and non-specific. If unexplained proteinuria accompanied by megaloblastic anemia and neurologic involvement are present, inherited metabolic diseases should be considered. Although the pathologic mechanisms were related to Cbl, the severity and prognosis of renal lesions were quite different. Molecular analysis is very important for the diagnosis, treatment and prognosis of such diseases.

## Data Availability

Data were collected from Children’s Hospital of Soochow University, Guangzhou Jinyu Medical Laboratory Center, Jiajian Check Medical Testing Corporation, and Beijing Zhiyin Oriental Conversion Medical Research Center. The datasets generated and/or analysed during the current study are available in the NCBI repository, [SRR12586543; SRR12586542; SRR12586541; SRR12586540.]. These materials described in the manuscript, including all relevant raw data are available upon request.

## Abbreviations

MMUT: Methylmalonate coenzyme A mutase
MMA: Methylmalonic acidemia
MMACHC: Methylmalonic aciduria type C with homocystinuria
AMN: Amnionless
Cbl: Cobalamin
CNCbl: Cyanocobalamin
AdoCbl: Adenosylcobalamin
MeCbl: Methylcobalamin
RBCs: Red blood cells
cblC: Methylmalonic acidemia and homocystinuria
T1WI: T1-weighted imaging
MRI: Magnetic resonance imaging
MMF: Mycophenolate mofetil
IGS: Imerslund–Gräsbeck syndrome
CUBN: Cubilin
IFD: Intrinsic factor deficiency
HUS: Hemolytic uremic syndrome
TMA: Thrombotic microangiopathy
